# Human infections due to *Salmonella* Blockley, a rare serotype in South Africa: a case report

**DOI:** 10.1186/1756-0500-5-562

**Published:** 2012-10-10

**Authors:** Thandubuhle Gonose, Anthony M Smith, Karen H Keddy, Arvinda Sooka, Victoria Howell, Charlene Ann Jacobs, Sumayya Haffejee, Premi Govender

**Affiliations:** 1 Centre for Enteric Diseases (CED), National Institute for Communicable Diseases (NICD) of the National Health Laboratory Service (NHLS), Johannesburg, South Africa; 2 Faculty of Health Sciences, University of the Witwatersrand, Johannesburg, South Africa; 3 Department of Microbiology, Greys Pathology Laboratory, NHLS, Pietermaritzburg, South Africa; 4 Division of Surveillance, Outbreak Response and Travel Health, NICD, NHLS, Johannesburg, South Africa; 5 KwaZulu Natal Department of Health, Pietermaritzburg, South Africa; 6 Centre for Enteric Diseases, National Institute for Communicable Diseases, Private Bag X4, 2131, Gauteng, Sandringham, South Africa

**Keywords:** Human infections, *Salmonella* Blockley, Rare serotype

## Abstract

**Background:**

Infections due to nontyphoidal *Salmonella* have increased worldwide over the last couple of decades. *Salmonella enterica* serotype Blockley (*Salmonella* Blockley) infections is associated with chickens and is a rarely isolated serotype in human infections in most countries.

**Case presentation:**

We report a case of human infections due to *Salmonella* Blockley in KwaZulu-Natal, South Africa in 2011. Three African males (aged 4, 14 and 16) presented to a clinic with diarrhoea, stomach cramps and headache. They started experiencing signs of illness a day after they consumed a common meal, consisting of meat, rice and potatoes. Stool specimens from the patients cultured *Salmonella* Blockley. The strains showed an indistinguishable pulsed-field gel electrophoresis pattern.

**Conclusion:**

This is the first recorded case of human infections due to *Salmonella* Blockley in South Africa.

## Background

Human infections due to *Salmonella* is a public health problem both in developed and in developing countries [1]. Annually, 1.6 million cases of typhoid fever, 1.3 billion cases of gastroenteritis and 3 million deaths due to *Salmonella* are reported worldwide [2]. Infections due to nontyphoidal *Salmonella* have increased worldwide over the last couple of decades and are the cause of acute gastrointestinal illness which is characterised by diarrhoea, stomach cramps and fever [3].

*Salmonella enterica* serotype Blockley (*Salmonella* Blockley) is a rarely isolated serotype in most countries. However, *Salmonella* Blockley was among the twenty most frequently isolated serotypes in 17 European countries over the period 1998 to 2002 [3]. Single outbreaks of *Salmonella* Blockley have been reported in the United States of America and Greece, while several sporadic cases have been reported in Europe [4,5]. In South Africa, only nine cases of *Salmonella* Blockley were identified by the Centre for Enteric Diseases (CED) of the National Institute for Communicable Diseases (NICD) over the period 2003 to 2011. We now report a case of human infections caused by *Salmonella* Blockley in KwaZulu-Natal, South Africa.

## Case presentation

Three African males from the same family (aged 4, 14 and 16 years) became ill after eating a meal on 23 September 2011. They presented to a clinic with diarrhoea, stomach cramps and headache on 24 September 2011, a day after consuming the meal. The meal consisted of meat, rice and potatoes. The meat was obtained from a cow that died on 22 September 2011; the cow died after it consumed an unspecified plastic material. The cow was subsequently slaughtered and the meat was shared among the villagers for consumption. According to the mother of the three children, the meat was prepared and boiled on the day of slaughter (22 September 2011), but they only warmed it up the following day when the children ate it. No other village members reported any symptoms of illness. On arrival at the clinic, the three patient’s temperature was checked and it was normal for the three of them. Stool samples were taken from the three patients and sent for laboratory for analysis. The patients were sent home with medication.

Identification of the isolates was done using VITEK-2 Compact (bioMérieux, Inc Durham NC, United States of America) and confirmed by serotyping using specific antisera (Mast Assure, Mast Group Ltd., Merseyside, United Kingdom), according to the Kauffman-White scheme. Antimicrobial susceptibility to ampicillin, augmentin, trimethoprim, sulphamethoxazole, chloramphenicol, nalixidic acid, ciprofloxacin, kanamycin, streptomycin, ceftriaxone, cefepime, imipenim and tetracycline was determined by Etest (bioMérieux, Marcy-l′Etoile, France). The genetic relatedness of the isolates was determined using pulsed-field gel electrophoresis (PFGE) analysis of *Xba*I-digested genomic DNA on a CHEF-DR III electrophoresis system (Bio-Rad Laboratories, Hercules, USA), using PulseNet protocol [6]. The patterns were analysed using BioNumeric (version 6.01) software (Applied Maths, Sint-Martens-Lartem, Belgium).

**Figure 1:**
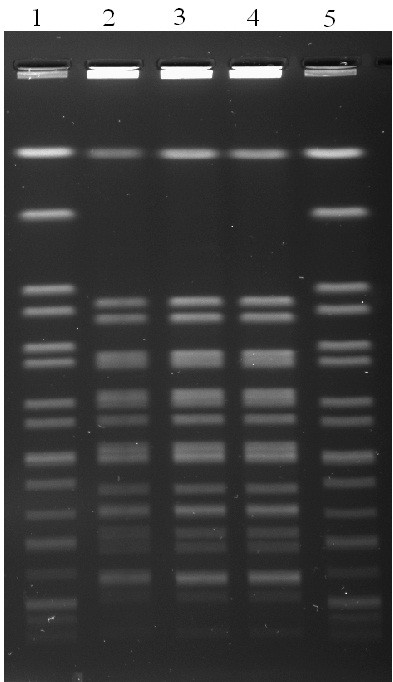
**PFGE analysis (*Xba*I digestion) of *Salmonella *Blockley isolates.** Lanes 2–4 are isolates from the three cases of foodborne illness; lanes 1 and 5 are the reference standard pattern (*Salmonella* Braenderup strain H9812).

Laboratory examination isolated *Salmonella* Blockley from the three stool samples. All the isolates were susceptible to ampicillin, chloramphenicol, streptomycin, sulphamethoxazole, trimethoprim, nalixidic acid; and all were resistant to tetracycline. PFGE restriction fragment patterns were identical for all three isolates (Figure 1). The identity in antibiotic susceptibility and PFGE patterns among the three *Salmonella* Blockley strains indicated that the source of infection was common. Unfortunately there were no food samples taken for analysis, therefore the source of infection remains unknown. Although chickens are the major source of *Salmonella* Blockley, previous studies have indicated that this serotype can be obtained from different sources such as eggs, smoked eel and vegetables [4,5]. Moreover, other factors such as food storage, unhygienic behavior of food handlers, poor food preparation and poor food serving can increase the risk of cross contaminations [4,5].

There are few reported cases of infections due to *Salmonella* Blockley. According to a PubMed literature search, the first reported cases date back to 1966 [5]. In 1989, the first isolation of *Salmonella* Blockley in Thailand from animal feed and chicken feathers was reported [7]. Recently, *Salmonella* Blockley isolation has been noted in other countries such as Europe and United States of America. However, it remains a rarely isolated serotype in human infections in South Africa and in most countries. Despite its low frequency of isolation, *Salmonella* Blockley has shown high rates of multidrug resistance among *Salmonella* isolates in Greece [8].

## Conclusions

To the best of our knowledge, this is the first documented case of human infections due to *Salmonella* Blockley in South Africa. Just like any other reported case of human infection due to *Salmonella* species, we emphasize that more awareness on hygiene, sanitation and proper preparation of food should be increased. Fortunately, the disease in this particular case was mild with no mortality occurring. Given the emergence of *Salmonella* Blockley in most countries, reported cases can be very useful in identifying the similarities between strains from different countries.

## Consent

Written informed consent was obtained from the patient’s parent/ legal guardian for publication of this Case report and any accompanying images. A copy of the written consent is available for review by the Editor of this journal.

## Abbreviations

DNA: Deoxyribonucleic acid; CHEF: Contour-clamped homogeneous electric fields; PFGE: Pulse-field gel electrophoresis.

## Competing interests

The authors declare that they have no competing interest.

## Authors’ contributions

TG, VH, SH carried out laboratory test for the identification of isolates. CAJ, PG participated in obtaining the information from family regarding food they ate and communicated it to laboratories. TG, AMS, KHK, AS participated in drafting the manuscript. All authors read and approved the final manuscript.

## Authors’ information

AMS – BSc (honours), PhD Microbiology; senior medical scientist at CED.

TG – BSc (honours) Microbiology; Intern medical scientist at CED.

## References

[B1] Niehaus JA Apalata T Coovadia MY Smith MA Moodley P An outbreak of foodborne Salmonellosis in rural Kwazulu-Natal South Africa Foodborne pathogens diseases 2011 8 693 697 10.1089/fpd.2010.0749 21388293

[B2] Pui CF Wong WC Chai LC Tunung R Jeyaletchumi P Noor Hidayah MS *Salmonella*: A foodborne pathogen Int Food Research J 2011 18 465 473

[B3] Kåre M Olsen JE Wegener HC Riemann HP, Cliver DO Salmonella infections Foodborne infections 2006 Third edition Elsevier Academic Press Publishers, California, USA 57 115

[B4] Fell G Hamouda O Lindner R Rehmet S Liesegand A Prager R An outbreak of Salmonella blockley infections following smoked eel consumption in Germany Epidemiol Infect 2000 125 9 12 10.1017/S0950268899004069 11057953 PMC2869563

[B5] Morse LJ Rubenstein AD A food-borne institutional outbreak of enteritis due to *Salmonella blockley* J Am Med Assoc 1967 202 939 940 10.1001/jama.1967.03130230065010 5630735

[B6] Ribot EM Fair MA Gautom R Cameron DN Hunter SB Swaminathan B Standardisartion of pulse-field gel electrophoresis protocols for subtyping for subtyping of Eschericia coli O157:H7, Salmonella, Shigella for PulseNet Foodborne Pathog Dis 2006 3 59 67 10.1089/fpd.2006.3.59 16602980

[B7] Bangtrakulnonth A Suthienkul O Kitjakara A Pornrungwong S Siripanichgon K First isolation of Salmonella Blockley in Thailand Southeast Asian J Trop Med Public Health 1994 25 668 692 7667714

[B8] Tassios PT Chadjichristodoulou C Lambiri M Kansouzidou-Kanakoudi A Sarandopoulou Z Kourea-Kremastinou J Molecular typing of multidrug-resistant Salmonella Blockley outbreak isolates from Greece Emerg Infect Dis 2000 6 60 64 10653572 10.3201/eid0601.000111 PMC2627977

